# Treatment persistence in patients with rheumatoid arthritis and ankylosing spondylitis

**DOI:** 10.1590/S1518-8787.2016050006265

**Published:** 2016-08-16

**Authors:** Marina Amaral de Ávila Machado, Cristiano Soares de Moura, Felipe Ferré, Sasha Bernatsky, Elham Rahme, Francisco de Assis Acurcio

**Affiliations:** I Programa de Pós-Graduação em Saúde Pública. Faculdade de Medicina. Universidade Federal de Minas Gerais. Belo Horizonte, MG, Brasil; II Research Institute of the McGill University Health Centre. Montreal, Quebec, Canada; III Division of Clinical Epidemiology. McGill University. Montreal, Quebec, Canada; IV Departamento de Farmácia Social. Faculdade de Farmácia. Universidade Federal de Minas Gerais. Belo Horizonte, MG, Brasil

**Keywords:** Arthritis, Rheumatoid, therapy, Spondylitis, Ankylosing, therapy, Antirheumatic Agents, therapeutic use, Biological Therapy, Medication Persistence

## Abstract

**OBJECTIVE:**

To evaluate treatment persistence in patients with rheumatoid arthritis and ankylosing spondylitis who started therapies with disease-modifying antirheumatic drugs (DMARD) and tumor necrosis factor blockers (anti-TNF drugs).

**METHODS:**

This retrospective cohort study from July 2008 to September 2013 evaluated therapy persistence, which is defined as the period between the start of treatment until it is discontinued, allowing for an interval of up to 30 days between the prescription end and the start of the next prescription. Odds ratio (OR) with 95% confidence intervals (95%CI) were calculated by logistic regression models to estimate the patients’ chances of persisting in their therapies after the first and after the two first years of follow-up.

**RESULTS:**

The study included 11,642 patients with rheumatoid arthritis – 2,241 of these started on anti-TNF drugs (+/-DMARD) and 9,401 patients started on DMARD – and 1,251 patients with ankylosing spondylitis – 976 of them were started on anti-TNF drugs (+/-DMARD) and 275 were started on DMARD. In the first year of follow-up, 63.5% of the patients persisted in their therapies with anti-TNF drugs (+/-DMARD) and 54.1% remained using DMARD in the group with rheumatoid arthritis. In regards to ankylosing spondylitis, 79.0% of the subjects in anti-TNF (+/-DMARD) group and 41.1% of the subjects in the DMARD group persisted with their treatments. The OR (95%CI) for therapy persistence was 1.50 (1.34-1.67) for the anti-TNF (+/-DMARD) group as compared with the DMARD group in the first year for the patients with rheumatoid arthritis, and 2.33 (1.74-3.11) for the patients with ankylosing spondylitis. A similar trend was observed at the end of the second year.

**CONCLUSIONS:**

A general trend of higher rates of therapy persistence with anti-TNF drugs (+/-DMARD) was observed as compared to DMARD in the study period. We observed higher persistence rates for anti-TNF drugs (+/-DMARD) in patients with ankylosing spondylitis as compared to rheumatoid arthritis; and a higher persistence for DMARD in patients with rheumatoid arthritis as compared to ankylosing spondylitis.

## RESUMO

**OBJETIVO:**

Avaliar a persistência do tratamento em pacientes com artrite reumatoide e espondilite anquilosante que iniciaram terapia com medicamentos modificadores do curso da doença (MMCD) e agentes bloqueadores do fator de necrose tumoral (anti-TNF).

**MÉTODOS:**

Este estudo de coorte retrospectiva de julho de 2008 a setembro de 2013 avaliou a persistência na terapia, definida como o tempo do início até a descontinuação, permitindo-se um intervalo de até 30 dias entre o fim da prescrição e o início da prescrição seguinte. *Odds ratio* (OR) com intervalos de confiança de 95% (IC95%) foram calculados por meio de modelos de regressão logística para estimar a chance de apresentar persistência na terapia após o primeiro e os dois primeiros anos de seguimento.

**RESULTADOS:**

Foram incluídos 11.642 pacientes com artrite reumatoide – 2.241 iniciaram uso de agentes anti-TNF (+/-MMCD) e 9.401 iniciaram MMCD – e 1.251 pacientes com espondilite anquilosante – 976 iniciaram uso de agentes anti-TNF (+/-MMCD) e 275 iniciaram MMCD. No primeiro ano de acompanhamento, 63,5% persistiram em terapia com anti-TNF (+/-MMCD) e 54,1% em uso de MMCD do grupo com artrite reumatoide. Em relação à espondilite anquilosante, 79,0% do grupo anti-TNF (+/-MMCD) e 41,1% do grupo MMCD persistiram no tratamento. O OR (IC95%) para persistência na terapia foi de 1,50 (1,34–1,67) para o grupo anti-TNF (+/-MMCD) comparado com MMCD no primeiro ano em pacientes com artrite reumatoide, e de 2,33 (1,74–3,11) em pacientes com espondilite anquilosante. Foi observada tendência semelhante ao final do segundo ano.

**CONCLUSÕES:**

Observou-se uma tendência geral de taxas mais elevadas de persistência na terapia com anti-TNF (+/-MMCD) em relação a MMCD no período estudado. Foram observadas taxas de persistência mais elevadas para os usuários de anti-TNF (+/-MMCD) em pacientes com espondilite anquilosante em relação a artrite reumatoide, e maior persistência para MMCD em pacientes com artrite reumatoide em relação à espondilite anquilosante.

## INTRODUCTION

The use of biological therapies deeply changed the treatment of rheumatoid arthritis (RA) and ankylosing spondylitis (AS), inflammatory rheumatic diseases that cause disabilities and affect patients’ functionality and quality of life. RA is characterized by symmetrical synovitis and AS affects the axial skeleton and causes lumbar pain and sacroiliitis^4^ ^,^ ^12^. Tumor necrosis factor blockers (anti-TNF drugs) are biological medications recommended for patients who still have high disease activity after undergoing first-line therapies, which comprise disease-modifying antirheumatic drugs (DMARD) for RA and nonsteroidal anti-inflammatory drugs (NSAID) for AS. DMARD are indicated for patients with AS in case of peripheral arthritis^5^ ^,^ ^18^ ^,^ ^23^ ^,^ ^25^.

In 1993, RA prevalence in Brazil was estimated to be 1.0%, with no more recent data having been published. AS prevalence in Latin America ranges from 0.30% to 0.19%, but it has not been determined for the Brazilian population^7^ ^,^ ^16^. In turn, RA and AS incidence rates are estimated to be 20-300/100,000 person-year and 0.4-7.3/100,000 person-year, respectively^27^ ^,^ ^a^.

In Brazil, the first anti-TNF agent (infliximab) was introduced in the Brazilian Unified Health System (SUS) for RA in 2002, and more recently (2010) for AS. Etanercept and adalimumab were introduced in the protocol of RA in 2006 and in the protocol of AS in 2010. DMARD and anti-TNF drugs are provided by SUS by its Specialized Component of Pharmaceutical Service, and its dispensation is entered in the outpatient information system (SIA) of DATASUS (Information Department of SUS), in its sub-system for authorization of high-complexity/high-cost procedures (APAC).

Despite the benefits anti-TNF drugs have in the control of both diseases^14^ ^,^ ^17^, some patients experience therapeutic failure or go by adverse events and end up interrupting their therapies^10^. However, no studies have been conducted so far to analyze the use of these medications in the context of SUS. To fill this knowledge gap, this study aimed to evaluate treatment persistence in patients with rheumatoid arthritis and ankylosing spondylitis who started therapies with DMARD and anti-TNF drugs.

## METHODS

A retrospective cohort study was conducted with data from DATASUS in Minas Gerais. Two administrative databases were used: SIA database, with APAC records from January 2008 to September 2013, which records the medications dispensed by SUS’ Specialized Component of Pharmaceutical Service; and the *Sistema de Informação sobre Mortalidade* (SIM – mortality information system) in the period between January 2006 to July 2011. The records of one same patient in these bases were linked by probabilistic-deterministic linkage, which enables to find different records of one same patient in distinct databases or in one same database^22^.

The study included patients diagnosed with RA and AS identified in the APAC/SIA database according to ICD-10 codes M45, M05, and M06. The first date of dispensations of DMARD or anti-TNF drugs was defined as the cohort entry; thus, only the patients who started on drug therapies from July 1, 2008 to June 31, 2013 were considered in the study. All patients were 18 years of age or older at the cohort entry, and were followed-up until the first of two dates: their date of death or the final date of the study (September 30, 2013).

Two exposure groups were considered according to the therapies dispensed at the cohort entry: anti-TNF drugs (+/-DMARD) and DMARD (monotherapy or association of two or more DMARD). The patients who started on anti-TNF drugs associated with DMARD at the cohort start were considered in the anti-TNF group. During the study, anti-TNF drugs infliximab, etanercept, and adalimumab and DMARD chloroquine, hydroxychloroquine, leflunomide, methotrexate, and sulfasalazine were available in SUS. Patients who used DMARD or anti-TNF drugs in the six months before the cohort entry were excluded from the DMARD group. The same way, the patients who used anti-TNF therapies six months before the cohort entry were excluded from the anti-TNF (+/-DMARD) group.

The patients were described according to their age at cohort entry, sex, and *per capita* income. The *per capita* income was obtained based on the linkage between the zip code available in the APAC/SIA database with the census areas used in 2010 census. The patients in the study were classified in eight income categories according to the criterion from Brazil’s federal government’s secretariat of strategic affairs^b^.

The use of medications was described for the first year and for the first two years of follow-up. A therapy was considered as discontinued after no drugs were dispensed in a period of 30 days following the end of a previous drug treatment prescribed, and treatment persistence was described as the period between the start and the discontinuation of a therapy. In the anti-TNF (+/-DMARD) group, the switch of an anti-TNF drug for another anti-TNF drug was considered to be a discontinuation of the initial therapy. We calculated the proportion of persistent patients by the first year by dividing the number of patients who persisted in their therapies for a year or longer by the number of patients who had a full year of follow-up or more. Similarly, we calculated the proportion of persistent patients by the first two years by the ratio between the number of patients who persisted in their therapies for two years or longer by the number of patients who had two years of follow-up or more. The one and two-year medication possession ratios (MPR) were obtained by dividing the total number of days of medication supply in one and two years by 365 and 730 days, respectively. Thus, the numerator corresponds to the total number of days of drug supply, regardless of whether there were intervals during dispensation events. The proportion of patients who started on anti-TNF therapies was also described for the DMARD group during use of DMARD or after DMARD discontinuation.

We calculated the frequency distributions for the categorical variables; and means and standard deviation (SD) or median and interquartile range (IQR) for the continuous variables. We calculated the difference between means and proportions among the groups in regards to the use of medications and respective 95% confidence intervals (95%CI). Besides that, we used Kaplan-Meier survival curve and log-rank test to compare therapy persistence rates among the studied groups. We used age, sex, and *per capita* income-adjusted logistic regression models to each disease to compare the proportion of persistent patients among the groups. We combined the income classes in the following way for this analysis: vulnerable class, lower middle class, and average middle class were considered as low income; upper middle class, lower upper class, and high upper class were considered as high income. A 5% significance level was adopted. The statistical analyses were conducted by SAS software, version 9.3 (SAS Institute Inc., Cary, NC).

This study was approved by the Research Ethics Committee of the Universidade Federal de Minas Gerais (UFMG - ETIC Process 0069.0.203.000-11). When applied, this article was written according to STROBE’s recommendations for cohort studies^15^.

## RESULTS

We included 12,893 patients in the cohort in total: 11,642 patients with RA and 1,251 patients with AS. Among the anti-TNF (+/-DMARD) group, most patients were started on therapies with adalimumab or etanercept, 23 patients were started on anti-TNF therapies associated with DMARD and, during the follow-up period, 103 patients were treated with the combined therapy. The groups of patients with RA were found to have higher age medians and higher frequency of females than the patients with AS. Most patients were classified as average middle class and upper middle class (Table 1).

**Table 1:** Baseline characteristics of patients with rheumatoid arthritis and ankylosing spondylitis who were included in the study according to their initial therapies.

Variable	Rheumatoid arthritis (N = 11,642)		Ankylosing spondylitis (N = 1,251)	

	DMARD	Anti-TNF drugs (+/-DMARD)	DMARD	Anti-TNF drugs (+/-DMARD)
			
	n = 9,401	n = 2,241	n = 275	n = 976
Age, median (IQR)	54 (45-62)	52 (42-61)	39 (30-48)	41 (32-50)
Sex (female , n (%))	7,820 (83.18)	1,680 (74.97)	106 (38.55)	328 (33.61)
*Per capita* income, n (%)*				
Extremely poor - up to R$81.00	0	0	0	0
Poor, but not extremely poor - up to R$162.00	0	0	0	0
Vulnerable- up to R$291.00	113 (1.32)	9 (0.44)	7 (2.75)	10 (1.11)
Lower middle class - up to R$441.00	934 (10.91)	134 (6.49)	39 (15.29)	63 (6.99)
Average middle class - up to R$641.00	3,319 (38.78)	785 (38.01)	93 (36.47)	284 (31.52)
Upper middle class - up to R$1,019.00	2,714 (31.71)	727 (35.21)	76 (29.80)	344 (38.18)
Lower upper class - up to R$2,480.00	1,264 (14.77)	317 (15.35)	36 (14.12)	161 (17.87)
High upper class - above R$2,480.00	215 (2.51)	93 (4.50)	4 (1.57)	39 (4.33)
Median (IQR), R$	619 (499-839)	655 (537-863)	573 (463-796)	659 (539-892)
Initial therapy				
Adalimumab (+/-DMARD)	-	1,050 (46.9)	-	512 (52.4)
Etanercept (+/-DMARD)	-	881 (39.3)	-	399 (40.9)
Infliximab (+/-DMARD)	-	310 (13.8)	-	65 (6.7)
Methotrexate (monotherapy)	1,699 (18.1)	-	19 (6.9)	-
Sulfasalazine (monotherapy)	279 (3.0)	-	256 (93.1)	-

DMARD: disease-modifying antirheumatic drugs; IQR: interquartile range

* 8.6% missing data.

More patients with RA were persistent in their anti-TNF therapies (+/-DMARD), after the end of the first year of follow-up, as compared with the DMARD group (95%CI 4.3–7.4% of the difference). Among the latter, 6.0% of the patients were started on anti-TNF therapies while using DMARD, and were considered to be persistent in their DMARD therapies. The therapy persistence and MPR means were also higher in the anti-TNF (+/-DMARD) group (95%CI 11–22 days of the difference; 95%CI 0.05–0.08 of the difference) (Table 2).

**Table 2:** Drug usage profile of patients with rheumatoid arthritis and ankylosing spondylitis.

First year of follow-up^a^	Rheumatoid arthritis		Ankylosing spondylitis	

	DMARD	Anti-TNF drugs (+/-DMARD)	DMA RD	Anti-TNF drugs (+/-DMARD)
			
	N = 7,883	N = 1,838	N = 236	N = 680
Therapy persistence				
Mean ± SD, days	286 ± 107	303 ± 99	249 ± 121	332 ± 76
Persistent patients, n (%)	4,269 (54.15)	1,167 (63.49)	97 (41.10)	537 (78.97)
Medication possession ratios (MPR), mean ± SD	0.64 ± 0.27	0.70 ± 0.26	0.56 ± 0.28	0.81 ± 0.21
Start of anti-TNF therapy				
During DMARD therapy, n (%)	470 (5.96)	-	27 (11.44)	-
After DMARD discontinuation, n (%)	211 (2.68)	-	14 (5.93)	-
Time to start anti-TNF therapy, median (IQR), days	183 (91-274)	-	184 (123-274)	-

First two years of follow-up^b^	DMARD	Anti-TNF drugs (+/-DMARD)	DMARD	Anti-TNF drugs (+/-DMARD)
	N = 6,102	N = 1,440	N = 179	N = 398

Therapy persistence				
Mean ± SD, days	431 ± 245	477 ± 246	357 ± 251	581 ± 216
Persistent patients, n (%)	1,799 (29.48)	557 (38.68)	36 (20.11)	232 (58.29)
Medication possession ratios (MPR), mean ± SD	0.57 ± 0.29	0.62 ± 0.29	0.43 ± 0.29	0.74 ± 0.26
Start of anti-TNF therapy				
During DMARD therapy, n (%)	677 (7.26)	-	31 (11.27)	-
After DMARD discontinuation, n (%)	488 (5.62)	-	24 (9.72)	-
Time to start anti-TNF therapy, median (IQR), days	306 (153-488)	-	259 (153-381)	-

DMARD: disease-modifying antirheumatic drugs; MPR: medication possession ratio; SD: standard deviation; IQR: interquartile range

^a^ Patients who had been followed up for less than a year were excluded from the analysis.

^a^ Patients who had been followed up for less than two years were excluded from the analysis.

Among the patients with AS, the persistence ratio was higher in the anti-TNF (+/-DMARD) group as compared to the DMARD group in the first year of follow-up (95%CI 27.5–40.5% of the difference). The therapy persistence and MPR means were also higher for the anti-TNF drug (+/-DMARD) users (95%CI 10–97 days of the difference; 95%CI 0.22–0.29 of the difference) (Table 2).

At the end of the second year and among the patients with RA, the same trend was observed (95%CI 4.6–6.6% of the persistence rate difference; 95%CI 31–60 day of the persistence mean difference). Among the patients with AS, also, more patients in the anti-TNF (+/-DMARD) group persisted in their therapies after the two first years (95%CI 26.0–39.7% of the persistence rate difference; 95%CI 185–265 day of the persistence mean difference) (Table 2).

Regarding the patients with RA, the AS group was found to be more persistent in their anti-TNF therapies (+/-DMARD) in the first and in the first two years of follow-up (95%CI 10.5–17.4% of the difference in the rate of persistent patients in the first year, and 95%CI 10.5–17.5% at the end of the second year). On the other hand, we observed a higher persistence rate for DMARD in patients with RA as compared with AS (95%CI 0.7–2.2% in the first year, and 95%CI 0.4–2.0% at the end of the second year). The survival curves of drugs for two year-follow up show the differences found in therapy persistence among groups (Figure, log-rank test, p < 0.0001). In the logistic regression models, the anti-TNF (+/-DMARD) group was found to be twice as likely to be therapy persistent than the DMARD group in the first year and in the first two years of follow-up considering patients with AS, whereas the increased persistence likelihood for patients with RA was 50.0% among the two groups. Among the patients with RA, the subjects who earned lower incomes were more likely to be persistent in the first year and in the first two years, and the male sex was associated with higher persistence in the first year (Table 3).

**Figure:**
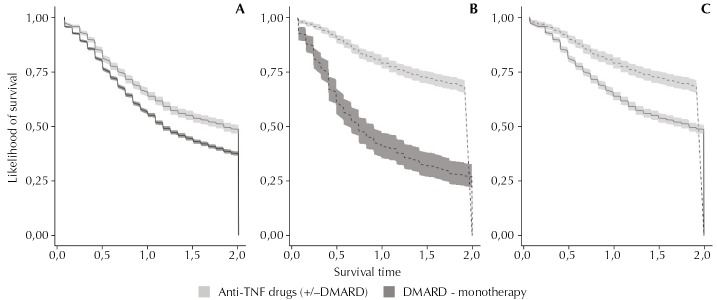
Survival curves of the medications.

DMARD: disease-modifying antirheumatic drugs

(A) Patients with rheumatoid arthritis.

(B) Patients with ankylosing spondylitis.

(C) Patients with rheumatoid arthritis (continuous line) and ankylosing spondylitis (dashed line) who used anti-TNF drugs (+/-DMARD).

Log-rank test: p < 0.0001.

**Table 3:** Factors associated with therapy persistence: logistic regression models.

Variable	Rheumatoid arthritis				Ankylosing spondylitis			

	First year of follow-up^a^		First two years of follow-up^b^		First year of follow-up^a^		First two years of follow-up^b^	

	Adjusted OR	95%CI	Adjusted OR	95%CI	Adjusted OR	95%CI	Adjusted OR	95%CI
Age	1.01	1.01–1.01	1.01	1.01–1.01	0.99	0.98–1.00	-	-
Male sex	1.14	1.02–1.27	-	-	-	-	-	-
Low income^c^	1.12	1.03–1.22	1.12	1.01–1.24	-	-	-	-
Anti-TNF therapy (+/-DMARD)	1.50	1.34–1.67	1.57	1.39–1.78	2.33	1.74–3.11	1.98	1.33–2.93

DMARD: disease-modifying antirheumatic drugs

^a^ Patients who had been followed up for less than a year were excluded from the analysis.

^a^ Patients who had been followed up for less than two years were excluded from the analysis.

^c^ Vulnerable class, lower middle class, and average middle class were considered as low income. Upper middle class, lower upper class, and high upper class were considered as high income.

Among the anti-TNF (+/-DMARD) group with RA, the users of adalimumab and etanercept were found to have persistence rates of 67.2% and 65.8%; whereas 46.6% of the patients persisted in their therapies with infliximab in the first year (95%CI 10.9–21.8% for difference between adalimumab and infliximab, and 95%CI 10.3–21.8% for difference between etanercept and infliximab). In the second year, infliximab was found to have a lower persistence rate than the other anti-TNF drugs. In the group with AS, the same trend was observed, and, in the first year, 80.0% and 80.8% of the patients on therapies with adalimumab and etanercept were found to be persistent, as compared to 60.4% of the patients on infliximab (95%CI 2.9–21.7% for difference between adalimumab and infliximab, and 95%CI 3.8–24.6% for difference between etanercept and infliximab). Among the patients diagnosed with RA who only used methotrexate, 28.7% of them persisted after the first year of follow-up, and the therapy persistence mean was 232 (SD = 116) days. At the end of the second year of follow-up, there were 7.6% persistent patients and 283 (SD = 205) days of therapy persistence. In the AS group, 93.1% of the patients started on sulfasalazine alone and were found to have very similar persistence rates as compared to the patients in the DMARD group. In the first year, 40.7% of the patients were found to persist in their therapies, and the therapy persistence mean was 248 (SD = 120) days. After the second year, the persistence rate was found to drop to 19.3%.

## DISCUSSION

In this study, the patients with RA and AS on anti-TNF therapies (+/-DMARD) were found to have better treatment persistence after one and two years being monitored as compared to the ones who used DMARD. Persistence in the anti-TNF therapy (+/-DMARD) in the first year of follow-up was 66.0% and 80.0% for the patients with RA and AS, respectively; in turn, these rates were reduced to 41.0% and 60.0% at the end of two years of follow-up. Better persistence results for patients with RA were found in France, England, and Holland, ranging from 70.0% to 78.0% in the first year and from 42.0% to 62.0% in the second year^6^ ^,^ ^8^ ^,^ ^11^ ^,^ ^26^. Patients with AS in Austria, France, and Denmark were found to have persistence rates similar to the ones found in this study, ranging from 70.0% to 83.0% in the first year and from 54.0% to 74.0% in the second year^6^ ^,^ ^9^ ^,^ ^20^. Studies which used administrative databases in the USA also revealed lower one-year persistence indices for patients with RA and AS, ranging from 40.0% to 57.0%^3^ ^,^ ^21^. The anti-TNF (+/-DMARD) group of patients with AS was found to have better results than the patients with RA. Brocq et al.^6^ (2007) and Sciré et al.^24^ (2013) also observed the discontinuation risk of anti-TNF therapy to be smaller in patients with AS as compared with the ones with RA, even after controlled for age, sex, disease duration, comorbidities, concomitant use of DMARD, type of anti-TNF drug, and calendar year.

In both disease groups, the use of infliximab was less frequent than the use of other anti-TNF drugs, and a lower persistence rate was found for this medication as compared to other agents in the same class. Infliximab is administered as an infusion, which may jeopardize therapy adherence, as patients depend on the availability and access to another health care service besides their dispensation pharmacy. In regards to adalimumab and etanercept, the patients themselves can administer these drugs via intramuscular injections by using prefilled syringes. Besides that, a study conducted in Minas Gerais showed that the monthly cost of the AS therapy with infliximab is R$800.00 to R$1,300.00 more expensive than the one with adalimumab and etanercept^c^, which suggests these two drugs can be better therapeutic alternatives to patients.

Persistence in the use of DMARD in the first year of follow-up was 54.0% and 41.0% for the patients with RA and AS, respectively; in turn, these rates were reduced to 29.0% and 20.0% at the end of two years of follow-up. Besides that, few users in the DMARD group were started on anti-TNF therapies during the study. Lie et al.^13^ (2010) reported two year-survival rates for therapies with methotrexate in patients with psoriatic arthritis and RA of 65.0% and 66.0%, respectively. It is hard to explain the low persistence in DMARD therapies based on the data shown, once clinical data and disease duration information are not available. In the case of AS, DMARD therapy is mainly indicated for peripheral arthritis, which affects around 20.0% of the patients, which may justify the low use of DMARD in this group^4^ ^,^ ^5^.

Discontinuing the therapy may lead to increased disease activity, especially in the case of anti-TNF drugs that play an important role in the treatment of these diseases^2^. The Brazilian Biologic Registry reported that 50.0% of the patients (70.0% with RA and 14.0% with AS) interrupt their treatments due to loss of effectiveness, whereas 30.0% of the discontinuation cases resulted from adverse events^28^. Adherence then becomes an important strategy to maximize pharmacotherapy results and to reduce possible damage related to extra-joint manifestations and loss of function and quality of life, besides avoiding potential direct and indirect costs for public health care systems regarding the management of these conditions. Therefore, non-persistence to therapies is believed to be harmful for patients with RA and AS, besides representing extra costs to health care systems.

The results of this study must be considered in light of some limitations. Firstly, APAC/SIA database does not consider dispensation from private pharmacies, and, therefore, the use of medications observed in this study may be underestimated. Secondly, APAC/SIA database has an invoicing nature and records the production and payment of outpatient procedures, and, therefore, is scarce concerning clinical data. The possibilities that some records may be missing or that some problems exist in the encoding of procedure cannot be ruled out either. Another limitation regards to the fact that the SIM base that was available might have been smaller than the studied period. However, this fact is not believed to have substantially affected our results, as the cohort period was short to observe a high number of deaths in the studied sample. Other studies have indicated a potential usefulness of these databases to study the paths of Brazil’s health care system beneficiaries^1^. Therefore, this study is believed to properly assess the use of medications by patients treated by SUS in Minas Gerais.

The anti-TNF therapy (+/-DMARD), in patients with RA and AS, was found to have higher persistence and MPR rates as compared to DMARD. The global persistence rates were better in AS than in RA. Adalimumab and etanercept were found to have better persistence results than infliximab, for both diseases. MPR in patients on DMARD was relatively low. Generally speaking, persistence was observed to be reduced in the second year of follow-up.

## Footnotes

a World Health Organization. The burden of musculoskeletal conditions at the start of the new millennium: report of a WHO Scientific Group. Geneva: World Health Organization; 2003. (WHO Technical Report Series, vol 919)

b Brasil. Governo Federal. Presidência da República. Secretaria de Assuntos Estratégicos. Perguntas e respostas sobre a definição da classe média. [cited 2014 Oct 15]. Available from: http://www.sae.gov.br/site/?p=13431

c Machado MAA. Análise farmacoepidemiológica e farmacoeconômica do tratamento da espondilite anquilosante com os bloqueadores do fator de necrose tumoral (TNF) [these]. Belo Horizonte (MG): Faculdade de Medicina, Universidade Federal de Minas Gerais; 2015.
